# Humanization of the *rpb9* Locus in Fission Yeast Reveals Conserved and Divergent Roles of *rpb9* and Human *POLR2I*

**DOI:** 10.3390/genes17060606

**Published:** 2026-05-27

**Authors:** Jared M. Finkel, Micah G. Williams, Mamta B. Nirmal, Samakshi Pandey, Erik D. Howe, Cameron T. Liu, Jeremy R. Lohman, Nimisha Sharma, Tommy V. Vo

**Affiliations:** 1 Department of Biochemistry and Molecular Biology, Michigan State University, East Lansing, MI 48824, USA; finkelja@msu.edu (J.M.F.); mgrant044@gmail.com (M.G.W.);; 2 Summer Research Opportunities Program (SROP), Michigan State University, East Lansing, MI 48824, USA; 3 University School of Biotechnology, Guru Gobind Singh (G.G.S.) Indraprastha University, Sector 16C, Dwarka, New Delhi 110078, India; samakshipandey06@gmail.com (S.P.);

**Keywords:** Pol II, *rpb9*, *POLR2I*, *S. pombe*, human, gene complementation, facultative heterochromatin, 6-azauracil, 5-fluorouracil

## Abstract

**Background/Objectives**: RNA polymerase II is a multifunctional complex that is critical for gene regulation and environmental responses. Its POLR2I subunit in humans is associated with various pathologies, including cancer chemoresistance. However, much of our understanding of how POLR2I functions is inferred from studies of its homologs in yeasts called Rpb9. Here, we endogenously humanized the *rpb9* gene of the fission yeast *Schizosaccharomyces pombe* to examine the functional capabilities of POLR2I. **Methods**: We edited the genomic *rpb9* locus in *S. pombe* so that it encodes the human POLR2I protein, and investigated functional and structural conservation. **Results**: With our humanized yeast system, we find widespread functional complementation by human *POLR2I* of *S. pombe rpb9* roles in yeast growth, chronological aging, and stress responses. We also find that *POLR2I* complements novel roles for yeast *rpb9* in facultative heterochromatin assembly, resistance against the chemotherapy 5-fluorouracil, and resistance against the fungicide thiabendazole. In contrast, we find that *POLR2I* cannot complement the role of *rpb9* in resistance against the transcription elongation inhibitor 6-azauracil (6-AU) in our system. Interestingly, *POLR2I* could complement 6-AU resistance if ectopically expressed. Lastly, we observe extensive structural homology between Rpb9 and POLR2I proteins. **Conclusions**: Our study establishes an endogenous cross-species gene complementation strategy that uncovers both conserved and rewired functions of fission yeast *rpb9* and its human homolog, *POLR2I*. In addition to validating conserved roles, we also identified conservation of previously unrecognized roles of *rpb9* in heterochromatin formation and chemoresistance.

## 1. Introduction

Gene regulation is a fundamental process that shapes cell fate and behaviors. In eukaryotes, gene expression is prominently regulated at the level of chromatin through transcriptional [1], co-transcriptional [2], and post-transcriptional mechanisms [3]. DNA-dependent RNA polymerase II (Pol II) plays a central role in these processes by functioning both as an enzyme and as a molecular recruitment platform [4]. As an enzyme, Pol II mediates transcription by synthesizing RNA [5,6,7,8]. As a platform, it can accommodate diverse post-translational modifications and interact with other biomolecules to coordinate transcription, chromatin modifications, chromatin remodeling, and RNA processing [9,10,11].

Pol II comprises 12 core protein subunits that collectively mediate its distinct functions [12,13,14,15]. While most Pol II subunits are essential for viability in yeast and metazoans, the Rpb9 subunit (also known as POLR2I in humans) is non-essential but is involved in environmental responses [16,17]. Most of our understanding of the molecular functions of Rpb9 comes from studies in the model budding yeast *Saccharomyces cerevisiae*, which indicate that Rpb9 plays important roles in transcription start site selection, transcription elongation, transcriptional fidelity, and transcription-coupled DNA repair [18,19,20,21]. Rpb9 performs these roles, in part, by modulating interactions of Pol II with transcription factors such as TFIIS [20] and TFIIF [22]. In contrast, there is a paucity of studies that have directly interrogated the molecular functions of human POLR2I [23]. This limits our ability to directly translate knowledge of yeast Rpb9 to its human homolog POLR2I. As recent studies showed that POLR2I amplification or upregulation is associated with colorectal cancer metastasis [24], chemoresistance of head and neck cancer [25], and hypertensive nephropathy [26], elucidating its molecular roles could advance our understanding of the etiology of these conditions.

Cross-species gene complementation is a powerful genetic approach for assessing the functional conservation of homologous coding genes [27,28,29,30]. For a given gene pair in which one gene encodes a protein with established function(s) and the other encodes a homolog with unclear function(s), the better-characterized gene is replaced with the less-characterized counterpart [31]. Functional assays are then used to determine which known activities can be complemented by the uncharacterized homolog. Successful complementation provides strong genetic evidence that the two genes (likely via their encoded proteins) share the function(s) in question. Gene complementation studies have been conducted to test whether human *POLR2I* can complement the roles of *rpb9* in the model yeasts *S. cerevisiae* and *S. pombe*. However, the biological interpretation of some results has been challenging. For example, high-level expression of *POLR2I* suppressed the hypersensitivity of yeast lacking *rpb9* (*rpb9Δ*) to elevated temperature, whereas lower expression levels produced no complementation [17,32]. This makes it unclear whether yeast *rpb9* and human *POLR2I* genuinely share temperature-related roles or if the complementation is rather context-dependent. Such challenges stem from the design of previous gene complementation studies [31,33], which relied on ectopic expression of *POLR2I* from plasmids in *rpb9Δ* yeast cells, followed by phenotypic analysis [17,34]. Although this strategy has enabled powerful functional interrogation of human genes in genetically tractable yeast systems, it also introduces confounding variables, including disruption of native regulatory context, non-physiological expression levels (determined by plasmid copy number or artificial promoters), and dependence on selective pressures to maintain plasmids. Refining the design of gene complementation studies for *rpb9^POLR2I^* to minimize these extraneous variables would strengthen the biological interpretation of their functional relationships.

In this study, we developed a genomic gene complementation strategy to functionally re-examine human *POLR2I* in the fission yeast *S. pombe*. This approach overcomes limitations of traditional plasmid-based systems by directly replacing the endogenous *rpb9* gene with *POLR2I*, at the native *rpb9* locus, thereby preserving the native regulatory context of the *rpb9* locus. Genomic integration also enables analysis of *POLR2I* function under standard non-selective rich media conditions. Consistent with previous plasmid-based complementation studies [17,34], we find that endogenously expressed *POLR2I* complements *S. pombe rpb9* roles in cell growth in the presence of several stress conditions. In addition, we identify a previously unrecognized and conserved role for *rpb9^POLR2I^* in the formation of facultative heterochromatin in *S. pombe*. Notably, our system reveals a lack of complementation in response to 6-azauracil (6-AU), a transcription elongation inhibitor, suggesting partial functional divergence between fission yeast *rpb9* and human *POLR2I*. Collectively, our study establishes a physiologically relevant gene complementation framework that both reproduces and extends the current understanding of *POLR2I* function.

## 2. Materials and Methods

### 2.1. Yeast Culturing and Manipulation

The *S. pombe* yeast strains used in this study are listed in Table S1. The *rpb9Δ* yeast strain was constructed by replacing the *rpb9* open reading frame with the *kanMX* gene by using a PCR-based gene deletion procedure with transformation by the lithium acetate method, as previously described [35]. The *kanMX* gene was derived from a pFA6a-*kanMX* plasmid that was purchased from Addgene (Addgene 39296; Watertown, MA, USA). All yeast strains were cultured in media that were based on the yeast extract rich medium with glucose and adenine supplement (YEA; 30 g/L glucose, 5 g/L yeast extract, 75 mg/L adenine, pH 5.5) and incubated at 32 °C, unless noted otherwise. To make plates, YEA-based media was prepared with 2% agar. Liquid cultures were grown at 32 °C with 220 rpm shaking for aeration. For our experiments, we used YEA media with the following: 5-FOA (GoldBio, cat no. F-230, 850 mg/L, St. Louis, MO, USA), G418 sulfate (GoldBio, cat no. G-418, 50 mg/L), NaCl (Sigma, cat no. 746398; St. Louis, MO, USA), 5-fluorouracil (Sigma, cat no. F6627, 25 μM), thiabendazole (Fisher Scientific, cat no. AAJ6000909, 20 μg/mL; Waltham, MA, USA), lithium chloride (Sigma, cat no. L7026), and 6-azauracil (Fisher Scientific, cat no. A05585G). For experiments using cells that carried ectopic expression plasmids, cell manipulation and spotting assays were performed as previously described in [17].

### 2.2. Endogenous Humanization of rpb9 in S. pombe

To integrate either *POLR2I* or *rpb9* into the endogenous *rpb9* locus, we started with a wild-type yeast strain that has an intact *rpb9+* locus and lacks the *ura4* and *kanMX* genes. First, we constructed a pFA6a-*ura4*-*kanMX* plasmid by cloning the *ura4* gene, along with 500 bp upstream and downstream regions, into the commercial pFA6a-*kanMX* plasmid (Addgene 39296). The upstream and downstream regions include the native ura4 promoter and termination sequences [36]. PCR amplification of the *ura4* locus was performed to add AscI and BglII restriction enzyme sites onto the PCR amplicon ends. Subsequently, conventional restriction enzyme cloning was performed to integrate the *ura4* amplicon into pFA6a-*kanMX*, placing it upstream of the *kanMX* promoter. The resultant pFA6a-*ura4*-*kanMX* plasmid was verified by Sanger sequencing.

Next, PCR-based homologous recombination [35] was used to replace the endogenous *rpb9* open reading frame DNA sequence with *ura4*-*kanMX*. The *ura4-kanMX* cassette was PCR-amplified using Splicing by Overlapping Extension (SOE) PCR [37] to append ~300 bp of sequence homology to the genomic region that flanks the *rpb9* open reading frame. Cells, where the swap was successful, grew on Pombe Minimum Glutamate (PMG) media lacking uracil and on YEA media with G418, but failed to grow on YEA media with 5-FOA.

Finally, PCR-based homologous recombination [35] was used to replace the *ura4-kanMX* cassette with the open reading frame DNA sequence of *POLR2I* or *rpb9*. The *POLR2I* DNA sequence was codon-optimized for *S. pombe* and synthesized as a double-stranded DNA product by the Integrated DNA Technologies (IDT) company. The *rpb9* DNA sequence was PCR-amplified from the ancestral wild-type strain TVTV39. Cells that successfully lost the *ura4-kanMX* cassette grew on YEA media with 5-FOA, but failed to grow on PMG media lacking uracil and YEA media with G418. The genotypes of the resultant yeast strains were verified by PCR and Sanger sequencing analyses. The DNA sequence of the *POLR2I* open reading frame used in this study is provided in Table S2.

### 2.3. Yeast Spotting Assays

Yeast cells were pre-cultured at 32 °C for 220 rpm overnight until cells reached the log growth phase. Afterwards, cell optical densities (OD_600_) were measured using a Denovix DS-11+ apparatus (Denovix; Wilmington, DE, USA), and then cells were normalized to the equivalent OD_600_ of 0.2–0.5. Finally, cells were serially diluted 4-fold or 10-fold, and equal volumes of cells were spotted onto the indicated media plates. Spotted plates were incubated at 32 °C without shaking for 2–5 days. Images were collected using a Bio-Rad Chemidoc imager (Bio-Rad; Hercules, CA, USA) using the Colorimeteric setting. Any image correction was uniformly applied to the entirety of the shown images.

### 2.4. Western Blotting

Western blotting was essentially performed as previously described [38]. Specifically, approximately two OD_600_ units of yeast cells were harvested by centrifugation in 2 mL screw-cap tubes. The cell pellet was resuspended in 200 µL of cold 20% trichloroacetic acid (TCA). Approximately 400 µL of acid-washed glass beads were added, and the cells were lysed using mechanical disruption with a bead beater (20 s beating × 3 cycles with 45-s intervals on ice). Following lysis, each tube was punctured, placed in a 5 mL polystyrene collection tube, and the lysate was collected by centrifugation at 1000 rpm for 30 s. The remaining beads were washed with 400 µL of cold 5% TCA, spun again, and the wash was pooled with the initial lysate. The combined lysate (~600 µL total) was transferred to a 1.5 mL microcentrifuge tube and centrifuged at 13,000 rpm for 5 min at 4 °C to pellet the proteins. The supernatant was discarded, and the remaining protein pellet was centrifuged 2–3 times, followed by removal of any residual TCA. The resulting protein pellet was resuspended in 100 µL of SDS-PAGE 1× sample loading buffer (Fisher Scientific, cat no. NP0007-2988243). The samples were then boiled at 95 °C and loaded on SDS-PAGE 4–12% Bis-Tris gels (Fisher Scientific, cat no. 25010870). Electrophoresis was performed in an Invitrogen mini tank apparatus using NuPage 1× running buffer (Fisher Scientific, cat no. NP0001) at room temperature at 200 volts for 45 min. Proteins were transferred onto PVDF membranes (pre-activated with methanol) using wet transfer (Nupage 1× transfer buffer (NP0006-2988243) at room temperature.

Then, membranes were blocked for 1 h at room temperature in 1× TBST (Tris-buffered saline with 0.1% Tween-20) or 1× PBST (phosphate-buffered saline with 0.1% Tween-20) containing 5% non-fat dry milk. Membranes were then incubated for 1 h at 37 °C with 1:2000 mouse monoclonal anti-GFP (Sigma, cat no. 11814460001, clones 7.1 and 13.1) or 1:1000 mouse monoclonal anti-tubulin (Sigma, cat no. T5168) that was diluted in blocking buffer. After washing three times with 1× TBST or 1× PBST (10 min each), membranes were incubated with an HRP-conjugated secondary anti-mouse (Jackson laboratory, cat no. 115-035-146, at 1:5000 dilution) for 1 h at room temperature. Following three additional 1× TBST or 1× PBST washes, signal detection was performed using an enhanced chemiluminescence (Cytiva, cat no. RPN2232-18206643; Wilmington, DE, USA) substrate and visualized using the Bio-Rad Chemidoc imaging system (Bio-Rad). 

### 2.5. Live-Cell Microscopy

To visualize Rpb9 or POLR2I localization in yeast cells, we used strains where the *gfp* gene was genomically integrated adjacent to the *rpb9* or *POLR2I* open reading frames to encode for C-terminally tagged Rpb9-GFP or POLR2I-GFP fusion proteins. Yeast cells were grown in YEA media to the exponential growth phase, and then 1 OD_600_ of cells were washed with phosphate-buffered saline (PBS) by pelleting cells in a centrifuge at ≥13,000× *g* for 1 min, repeated three times. To visualize nuclear DNA, cells were stained with Hoescht 33342 by adding the dye to create a 1 μg/mL solution. Cells were then incubated in darkness for 15 min. Next, cells were added to a glass slide and visualized using the Brightfield, DAPI, or eGFP fluorescence settings of the ZEISS Axio Observer Z1 inverted fluorescence microscope using the 63x oil immersion objective. Images were processed using the ZEN Pro (ZEISS) software (version 2.0).

### 2.6. Chronological Aging Assay

Yeast cells were cultured in YEA liquid media at a constant 1:5 volume-to-flask maximum volume ratio, at 32 °C with 220 rpm shaking for aeration. To assess the chronological lifespan of the various yeast strains, cultures were left shaking continuously without changing the media. Day-0 is defined as the day when cultures were in an exponential growth phase. Day-1 is defined as the start of the stationary phase. Day-1+ is defined as the days after initially reaching the stationary phase. On various days, cells were normalized by OD_600_ across strains, then serially diluted 1:4, and finally spotted onto YEA media plates. Plates were incubated at 32 °C for 3 days to assess strain viability based on yeast growth patterns.

### 2.7. Chromatin Immunoprecipitation

Chromatin immunoprecipitation was mostly performed as previously described [38]. Specifically, *S. pombe* cells were inoculated in 50 mL of YEA medium at 32 °C with shaking at 220 rpm to an OD_600_ of 0.5–0.6. Cells were crosslinked with 1% formaldehyde for 20 min at room temperature, followed by quenching with 2.5 M glycine. Next, cells were washed twice with 20 mL of cold 1× PBS. The cell pellet was resuspended in 350 µL of ChIP lysis buffer (50 mM HEPES, pH 7.5, 140 mM NaCl, 1 mM EDTA, 1% Triton X-100, 0.1% sodium deoxycholate, EDTA-free 1X cOmplete) and disrupted with 1 mL of glass beads using bead-beating (1 min beating, 2 min on ice, repeated 3 times). Chromatin was sheared using a QSonica Q800R sonicator (20 sec ON/40 sec OFF cycles, 70% amplitude) for 10 min at 4 °C. Cell debris was removed by centrifugation at 4 °C, 1500× *g*, for 5 min. The supernatant was transferred to fresh 1.5 mL tubes and diluted with ChIP lysis buffer. Lysates were pre-cleared with 20 µL of protein A/G agarose beads (Santa Cruz, cat no. A3124; Dallas, TX, USA) and incubated at 4 °C for 1 h. An amount of 50 µL of pre-cleared lysate was reserved as the 5% input control. The remaining lysate was incubated with the H3K9me2 (Abcam, cat no. ab115159; Waltham, MA, USA) antibody and protein A/G agarose beads overnight at 4 °C. Beads were washed sequentially (twice each) with the following buffers: ChIP Buffer I (50 mM HEPES, pH 7.5, 140 mM NaCl, 1 mM EDTA, 1% Triton X-100, and 0.1% deoxycholate), ChIP Buffer II (same as Buffer I, but with 500 mM NaCl), ChIP Buffer III (10 mM Tris-HCl, pH 8.0, 250 mM LiCl, 0.5% NP-40, 0.5% deoxycholate, and 1 mM EDTA), and 1× Tris-EDTA buffer. 

Immunoprecipitated chromatin was extracted by incubating the agarose beads with 100 µL of elution buffer (50 mM Tris-HCl, pH 8.0, and 10 mM EDTA) at 65 °C and agitating at 1000 rpm for 30 min. This step was repeated to obtain a total volume of 200 µL eluent. To each ChIP sample, 4 µL of 5N NaCl and 1 µL of RNase A (Fisher Scientific, 20 mg/mL) were added, and samples were incubated overnight at 65 °C in a water bath to reverse crosslinks. Input samples were adjusted to 200 µL with elution buffer, followed by the addition of 2.6 µL of 5N NaCl and 1 µL of RNase A, and incubated overnight at 65 °C in a water bath to reverse crosslinks. Afterwards, 1 µL of Proteinase K (Fisher Scientific, 20 mg/mL) was added to each tube and incubated at 50 °C for 1 h. Finally, DNA was purified by ethanol precipitation using 3 M sodium acetate and 1 µL glycoblue (Fisher Scientific, 15 mg/mL), and then resuspended in 50 µL of molecular-grade water. For quantitative PCR (qPCR), 1 µL of ChIP DNA was used per reaction with a Power SYBR Green PCR master mix (Life Technologies, cat no. 4367659; Waltham, MA, USA). Oligos used for PCRs are listed in Table S3.

### 2.8. RNA RT-qPCR

Total RNA was isolated from 10 mL of *S. pombe* cells that were grown in liquid YEA media at 32 °C to OD_600_ between 0.5 and 1. Cells were pelleted by centrifugation at 4000× *g* for 5 min at 4 °C, with the media discarded afterwards. AES buffer (50 mM sodium acetate, pH 5.3, 10 mM EDTA, and 1% SDS) was added to the cell pellet and mixed gently. In a fume hood, acidic phenol (pH 4.5) was added to the cell suspension and gently mixed by inverting the tube 10 times. Next, the sample was vortexed for 10 sec and incubated in the 65 °C water bath for 1 min. This vortex and incubation cycling was repeated five times without interruption. The resulting cell–AES–phenol mixture was centrifuged at 4000× *g* for 5 min at 6 °C. The aqueous phase was transferred to a new tube, then phenol–chloroform–isoamyl alcohol (25:24:1) (VWR) was added to the sample, and the tube was inverted gently 10 times to mix, followed by centrifugation at 4000× *g* for 5 min at 6 °C. The aqueous phase was transferred to a new tube, and then chloroform was added to each sample, gently mixed by inversion (10 times), and centrifuged again at 4000× *g* for 4 min at 6 °C. Carefully, the aqueous phase was transferred to a new tube. To this, 3M NaOAc and glycoblue (Fisher Scientific, 15 mg/mL) were added and mixed thoroughly by inverting the tube 10 times. An equal volume of 100% isopropanol was then added, and the tube was inverted 10 times to mix. The sample was centrifuged at maximum speed for 20 min at 6 °C. A moderate RNA pellet was observed, the supernatant was discarded, and the pellet was washed with 900 µL of 75% ethanol by inverting the tube 10 times, followed by centrifugation at maximum speed for 5 min at 6 °C. The supernatant was removed using a P1000 pipette, and any residual liquid was removed using a P200 or P20 pipette. The pellet was air-dried for 5 min before adding 100 µL of DEPC-treated water. The sample was incubated at room temperature for 1 min to dissolve the pellet and mixed gently by pipetting. The RNA concentration was measured using a DS-11+ spectrophotometer (Denovix). Then, 10 µg of isolated RNA was treated with Turbo DNase (Fisher Scientific, cat no. AM2238) in 50 uL of total volume to remove genomic DNA. Next, the volume was brought to 100 uL by adding DEPC-treated water. 100 µL of phenol–chloroform–isopropyl alcohol was added. The sample was gently inverted 10 times to mix and centrifuged at 20,000× *g* for 5 min at 6 °C. An amount of 70 µL of the top aqueous layer was carefully transferred to a new microcentrifuge tube. To this, 7 µL of 3M sodium acetate and 1 µL of glycogen were added and mixed by gentle tapping. Then, 235 µL of 200-proof ethanol was added and mixed by inverting the tube 40 times. The tubes were incubated overnight at −20 °C to precipitate the RNA. The sample was centrifuged at maximum speed (20,000× *g*) for 30 min at 6 °C to pellet the purified RNA. After centrifugation, the supernatant was carefully discarded using a P1000 pipette, leaving behind the RNA pellet. To wash the pellet, 900 µL of 75% ethanol was added, and the tube was inverted 10 times to mix. The sample was then centrifuged again at 20,000× *g* for 5 min at 6 °C. The supernatant was removed. The remaining RNA pellet was air-dried for 5 min to allow the remaining ethanol to evaporate. Finally, 20 µL of DEPC-treated water was added to dissolve the RNA. The tube was incubated at room temperature for 1 min, mixed gently by pipetting, and the RNA concentration was measured. From the DNase-treated total RNAs, cDNA was synthesized using a high-capacity cDNA reverse transcription kit (Applied Biosystems, cat no. 4368814; Waltham, MA, USA). Quantitative PCRs (qPCRs) were performed using a Power SYBR Green PCR master mix (Applied Biosystems, cat no. 4367659).

### 2.9. Sequence Alignment Analyses

Gene sequences for *S. pombe rpb9* and human *POLR2I* were collected from Pombase and UCSC Genome Browser, respectively. Protein sequences for *S. pombe* Rpb9 were obtained from Pombase and human POLR2I was obtained from Uniprot. Visualization of gene sequence comparison and amino acid sequence comparison was generated using the Needleman–Wunsch algorithm from EMBOSS NEEDLE Pairwise Sequence Alignment (PSA). Values of sequence comparison for gene sequence and amino acid sequence were collected using the Clustal Omega tool from EMBL-EBI (version 1). For Figure S6, the protein sequences were aligned using ClustalW with the BLOSUM62 matrix.

### 2.10. Protein Structure Analyses

Empirical protein structures for *S. pombe* Rpb1, Rpb2, and Rpb9 were downloaded from the structures 3H0G-A, 3H0G-B, and 3H0G-I from the Protein Data Bank (PDB) database, respectively. The human POLR2I empirical structure was downloaded from 9EHZ-F from the PDB. AlphaFold-predicted structures of *S. pombe* Rpb9 and human POLR2I were downloaded from the AlphaFold database AF-O74635-F1 and AF-P36954-F1, respectively [39]. RMSD, TM-Score, and visualizations were determined by comparing structures using the Pairwise Structure Alignment tool from the PDB or PyMOL (version 3.1). To model the Rpb1-Rpb2-Rpb9 and Rpb1-Rpb2-POLR2I complexes, the AlphaFold server was used with the following sequences: *S. pombe* Rpb1 (UniProt P36594) truncated to reside at 1555, *S. pombe* Rpb2 (Q02061), and *S. pombe* Rpb9 (O74635) or human POLR2I (P36954). Three zinc ions were also included in the Rpb9 and POLR2I models.

## 3. Results

### 3.1. Swapping the Endogenous rpb9 Gene for PORL2I in the S. pombe Genome

**Figure 1:**
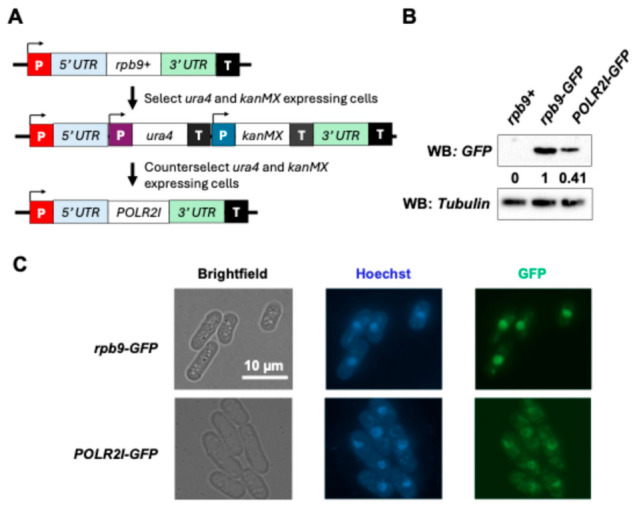
Genome-integrated *POLR2I* expresses nuclear-localized protein in *S. pombe*. (**A**) Illustration of our procedure to endogenously swap yeast *rpb9* for human *POLR2I*. (**B**) Western blot analysis of GFP-tagged endogenous Rpb9 or POLR2I protein in *S. pombe* cells. Wild-type cells without a GFP tag were used as a negative control. Levels of α-tubulin served as internal loading controls. Numbers indicate intensities of the GFP bands as percentages relative to the band of *rpb9*-GFP, which was defined as 1. (**C**) Brightfield and fluorescence microscopy of *S. pombe* cells with GFP-tagged Rpb9 or POLR2I protein. Hoechst stains nuclear DNA.

To humanize *rpb9* in *S. pombe* cells, we performed a two-step, scarless genome swapping procedure to replace the entire *rpb9* open reading frame (ORF) with an ORF corresponding to human *POLR2I* (Figure 1A). First, PCR-based homologous recombination was used to swap out the *rpb9* ORF for a *ura4-kanMX* DNA cassette. Then, another round of PCR-based homologous recombination was used to replace the *ura4-kanMX* cassette with the *POLR2I* ORF to generate the humanized yeast strain (*rpb9Δ::POLR2I*) (Table S2). The intermediatory *ura4* and *kanMX* genes permitted selection and counter-selection of our yeast constructs (Table 1).

Throughout this procedure, PCR was used to confirm successful completion of the two steps (Figure S1). We also validated the integration of *POLR2I* by Sanger sequencing. The humanized *rpb9Δ::POLR2I* yeast strain preserves the endogenous promoter, 5′- and 3′- untranslated regions (UTRs) at the native *rpb9* genomic locus. Effectively, we constructed a truly humanized *S. pombe* strain that carried a *POLR2I* ORF sequence in place of the *rpb9* ORF sequence, within the yeast genome, and that could be cultured in standard non-selective media.

The design of our cross-species gene complementation approach required two synthetic modifications of the *POLR2I* ORF. First, the *POLR2I* ORF had to be codon-optimized to ensure proper *POLR2I* expression in *S. pombe* cells, since codon biases differ between *S. pombe* and humans [42,43]. This differs from previous *rpb9^POLR2I^* complementation approaches that used *POLR2I* derived from human complementary DNAs (cDNAs) [17,32,34], which would not have been codon-optimized for *S. pombe* expression. Second, the *POLR2I* sequence in the *rpb9Δ::POLR2I* strain lacked introns. While *POLR2I* has five introns in human cells [44] and the general mechanisms of RNA splicing are well-conserved between *S. pombe* and humans [45,46], there are also key differences in the typical structure of introns from *S. pombe* and humans [47] that could make the splicing of human introns in *S. pombe* cells not ideal. This second modification was also adopted by previous *rpb9^POLR2I^* complementation studies [17,32,34]. Since our primary goal was to endogenously express the POLR2I protein in *S. pombe*, we decided to commercially synthesize double-stranded DNA that corresponded to codon-optimized intronless *POLR2I* and integrate this version into *S. pombe* cells (Table S2). The predicted POLR2I amino acid sequence is the same between our humanized yeast and human cells.

To measure the expression and localization of POLR2I protein in our humanized *S. pombe* cells, we first tagged the C-terminus of POLR2I with green fluorescent protein (GFP) and then performed Western blotting and fluorescence microscopy. As a control, we also generated *S. pombe* cells with endogenously tagged Rpb9-GFP at the C-terminus, to allow comparison of POLR2I-GFP expression with physiological levels of Rpb9 protein expression. We find that POLR2I-GFP is expressed, although the protein level is reduced by approximately 59% compared to Rpb9-GFP (Figure 1B). Also, both Rpb9-GFP and POLR2I-GFP primarily localize to the *S. pombe* nucleus (Figure 1C), as expected for core subunits of Pol II [15], although there was also a substantial cytoplasmic GFP signal for POLR2I-GFP (Figure 1C). These results indicate that *POLR2I*, which is genomically integrated at the yeast *rpb9* locus, is expressed in *S. pombe* cells to produce nuclear POLR2I protein that is markedly lower in level compared to Rpb9 in *S. pombe*.

### 3.2. Characterizing Effects of POLR2I on S. pombe Cell Growth in Non-Selective Media

**Figure 2:**
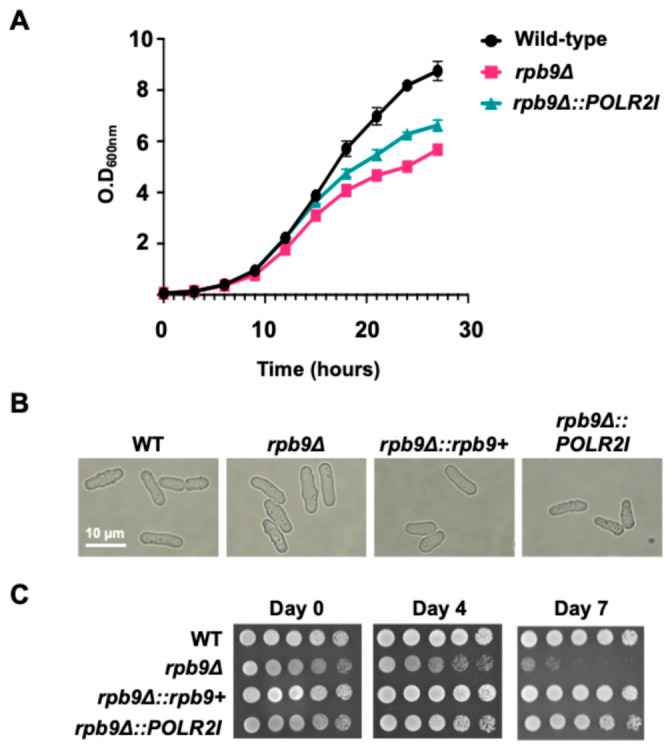
Effects of *rpb9* and *POLR2I* on yeast growth, physiology, and chronological aging. (**A**) Growth rate of yeast cultures as measured by optical density at 600 nm wavelength readings, N = 3 biological replicates. O.D_600_ data can be found in Table S4. (**B**) Brightfield microscopy images of cells belong to the indicated yeast strains. (**C**) Spotting assays showing the chronological aging phenotype of the indicated yeast strains, as a function of days after the yeast cultures had reached the stationary phase at 32 °C.

Hereafter, we performed our experiments using non-selective yeast extract with glucose and adenine supplement media (YEA), which is a standard rich media for *S. pombe* [48]. In this liquid media, we observed that *rpb9Δ::POLR2I S. pombe* cells grew substantially faster than *rpb9Δ* cells, but not fully as fast as wild-type (*rpb9+*) cells (Figure 2A). This growth pattern was also observed in the context of colony sizes where *rpb9Δ* cells often produced colonies that were smaller than wild-type ones, but *rpb9Δ::POLR2I* gave rise to colonies that were approximately as large as wild-type colonies (Figure S2). By light microscopy, we did not notice gross anomalies in the morphology of *rpb9Δ* or *rpb9Δ::POLR2I* cells (Figure 2B). These results suggest that endogenously expressed *POLR2I* in our humanization system can largely complement the general growth defects of *S. pombe* cells lacking *rpb9*.

We also examined whether *POLR2I* could complement the function of *rpb9* in *S. pombe* aging because a recent study indicated that *rpb9Δ* reduces the chronological lifespan of *S. pombe* cells [34]. Chronological lifespan is defined as the duration during which yeast cells remain viable in the stationary phase in unchanged liquid media (e.g., YEA) [49]. We tested whether *POLR2I* expression can restore the aging phenotype in *rpb9Δ* cells. We found that the viability of *rpb9Δ* cells was drastically reduced after 7 days of continuous culturing in unreplenished YEA media (Figure 2C). In contrast, cells where *rpb9+* or *POLR2I* was reintroduced into the native *rpb9* locus continued to grow robustly at the 7-day time-point (Figure 2C). These results agree with a prior study that *rpb9* loss reduces *S. pombe* chronological lifespan [34]. Furthermore, they indicate that human *POLR2I* complements the role of *S. pombe rpb9* in the chronological aging process.

### 3.3. POLR2I Complements Defects in Stress Responses in rpb9Δ Cells

**Figure 3:**
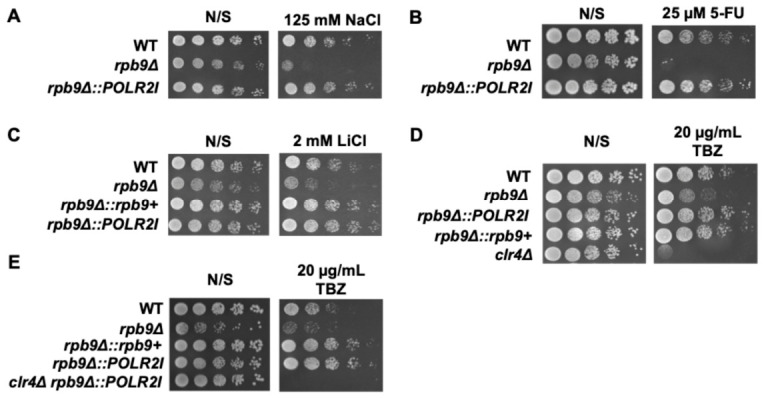
*POLR2I* complements certain *rpb9* roles in environmental stresses and facultative heterochromatin formation. (**A**) Spotting assays with serial dilutions of cells on non-selective (N/S) YEA media or YEA media with 125 mM NaCl. (**B**) Spotting assays with YEA media −/+ 25 μM 5-FU. (**C**) Spotting assays with YEA media −/+ 2 mM lithium chloride. (**D**,**E**) Spotting assays with YEA media −/+ 20 μg/mL TBZ. For all spotting assays, the yeast strains are indicated to the left of each panel. All incubations were performed at 32 °C.

In *S. cerevisiae* and *S. pombe*, *rpb9* was found to be important for yeast viability in response to various environmental stressors [16,17]. Additionally, ectopic high expression of *POLR2I* in *rpb9Δ* cells can complement environment-dependent growth defects [17,32]. We sought to test whether endogenously expressed *POLR2I* could complement similar stress-related growth defects. We found that, while 125 mM NaCl severely inhibits the growth of *rpb9Δ* cells on YEA media, growth was robustly restored for *rpb9Δ::POLR2I* cells (Figure 3A). We also tested whether *rpb9^POLR2I^* affects *S. pombe* response to the chemotherapy drug 5-fluorouracil (5-FU) because recent studies suggested that *rpb9* promotes the growth of *S. pombe* cells in the presence of 5-FU [50] and that *POLR2I* gene amplification might promote 5-FU resistance in human head and neck cancers [25]. We observed that *rpb9Δ::POLR2I* cells could grow on YEA media in the presence of 25 μM 5-FU, unlike *rpb9Δ* cells (Figure 3B). Additionally, a recent high-throughput phenomics study indicated that *rpb9Δ* cells are sensitive to environmental lithium chloride [51]. We were able to replicate this finding (Figure 3C). Interestingly, *POLR2I* expression was sufficient to restore growth of *rpb9Δ* cells in the presence of lithium chloride (Figure 3C). Lastly, we found that endogenous *POLR2I* rescued cell growth in the presence of the antifungal drug, thiabendazole (TBZ) [52,53] (Figure 3D). These various growth defects were also rescued by applying our humanization procedure to re-integrate *S. pombe rpb9* (*rpb9Δ::rpb9+*) (Figure 3C–E and Figure S3). Altogether, these results indicate that endogenous human *POLR2I* suppresses diverse environmental-related and *rpb9*-dependent yeast growth defects.

As *rpb9Δ* and *clr4Δ* cells are hypersensitive to YEA media containing the TBZ drug (Figure 3D) [54,55], we tested whether *rpb9* might mediate the yeast growth response to TBZ in a Clr4-dependent manner. To do this, we deleted the *clr4* gene in *rpb9Δ::POLR2I* cells and assayed their growth on YEA media with TBZ. Unlike *rpb9Δ::POLR2I* cells, we found that *rpb9Δ::POLR2I clr4Δ* cells failed to grow in the presence of TBZ (Figure 3E). This indicates that Rpb9 and POLR2I require Clr4 to promote yeast growth upon TBZ exposure.

### 3.4. Rpb9 and POLR2I Promote Facultative Heterochromatin Formation in S. pombe Cells

**Figure 4:**
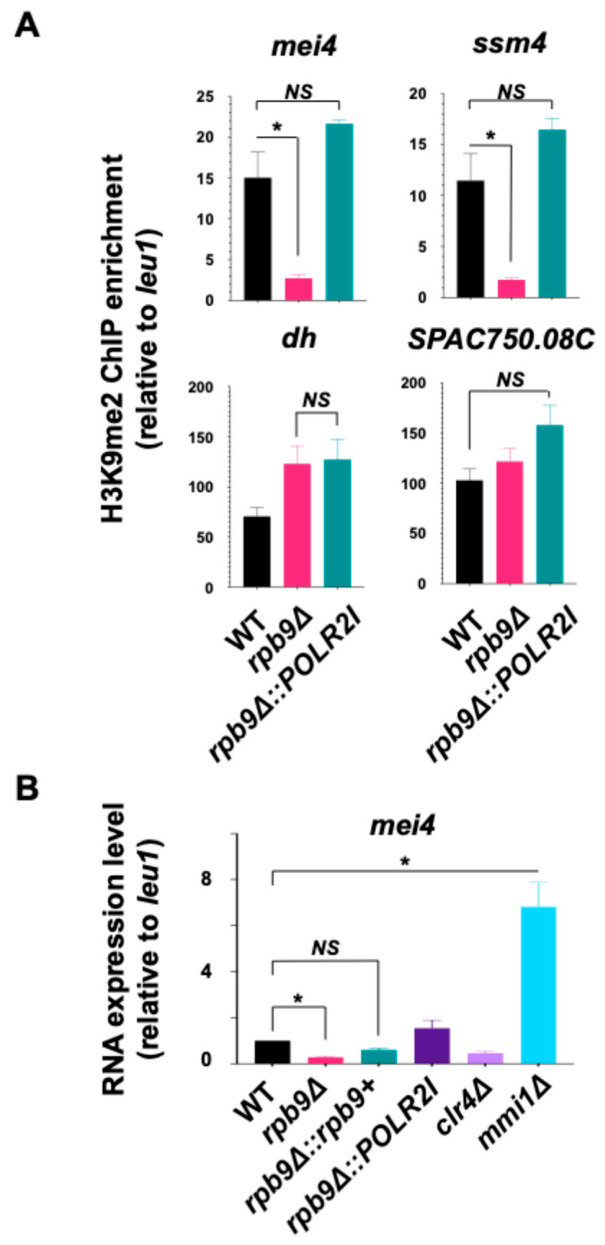
Rpb9 and POLR2I promote H3K9me2 at the Mmi1-dependent facultative heterochromatin islands *mei4* and *ssm4*. (**A**) H3K9me2 ChIP enrichments at the *mei4* and *ssm4* loci, at the pericentromeric *dh* repeats, and at the subtelomeric *SPAC750.08C* gene. (**B**) Fold-changes in steady-state RNA levels as determined by reverse transcription with quantitative PCR (RT-qPCR). Fold-changes are relative to wild-type, which is defined to be one. For both panels A and B, error bars denote the standard deviation of a representative ChIP experiment, N = 3. Statistical significance was determined by Student’s *t*-test. *NS* denotes not statistically significant. Asterisk (*) denotes statistical significance with *p*-value < 0.05.

We explored whether yeast *rpb9* has a novel role in the formation of facultative heterochromatin in *S. pombe*, which are repressed portions of the genome that are characterized, in part, by di-methylation of histone H3 at lysine-9 (H3K9me2) [56,57]. We previously found that *S. pombe* Rpb9 can directly interact with Mmi1 [58], which is required for H3K9me2 at some facultative heterochromatin regions [57]. Therefore, we tested whether *rpb9* was required for Mmi1-dependent H3K9me2 and whether *POLR2I* can complement that putative role. Using chromatin immunoprecipitation (ChIP), we found that *rpb9Δ* abolished H3K9me2 at the *mei4* and *ssm4* loci (Figure 4A), which are two representative genomic regions where Mmi1 promotes H3K9 methylation [57,59,60]. Furthermore, H3K9me2 levels were restored in *rpb9Δ::POLR2I* cells (Figure 4A). This indicates that *rpb9* and *POLR2I* may share a conserved mechanism that permits the formation of facultative heterochromatin in *S. pombe* cells.

The effects of *rpb9* on H3K9me2 were specific to the *mei4* and *ssm4* loci because H3K9me2 enrichment levels were neither reduced at pericentromeric *dh* repeats nor at the subtelomeric gene *SPAC750.08C* (Figure 4A). In wild-type *S. pombe* cells, the pericentromeres and subtelomeres are normally enriched for H3K9me2-marked heterochromatin [61]. The non-reduction in pericentromeric H3K9me2 suggests that the TBZ sensitivity of *rpb9Δ* cells might not be due to loss of heterochromatin assembly and impaired cohesion loading at centromeres [62,63]. Although *rpb9Δ* led to H3K9me2 loss at the *mei4* locus (Figure 4A), there was no accumulation of *mei4* RNAs (Figure 4B). Furthermore, the *mei4* RNA level did not increase in *clr4Δ* cells, where H3K9 cannot be methylated [64]. These *rpb9Δ* and *clr4Δ* results agree with conclusions from a previous study where *clr4* loss, alone, was insufficient to increase RNA from another Mmi1-regulated locus [57]. Repression of these loci is possibly enforced by two parallel mechanisms of Clr4- and Rpb9-dependent H3K9 methylation, and via RNA degradation that is mediated by the polyA-binding protein Pab2 and the nuclear exosome [57,65,66,67]. Supporting this, we found that *mei4* RNA is significantly increased upon the loss of Mmi1 (Figure 4B), which is required for both H3K9me2 and RNA degradation at the *mei4* locus.

### 3.5. Endogenously Expressed POLR2I Does Not Complement Growth Defects of rpb9Δ Cells Under 37 °C Heat Stress or in the Presence of 6-Azauracil (6-AU)

**Figure 5:**
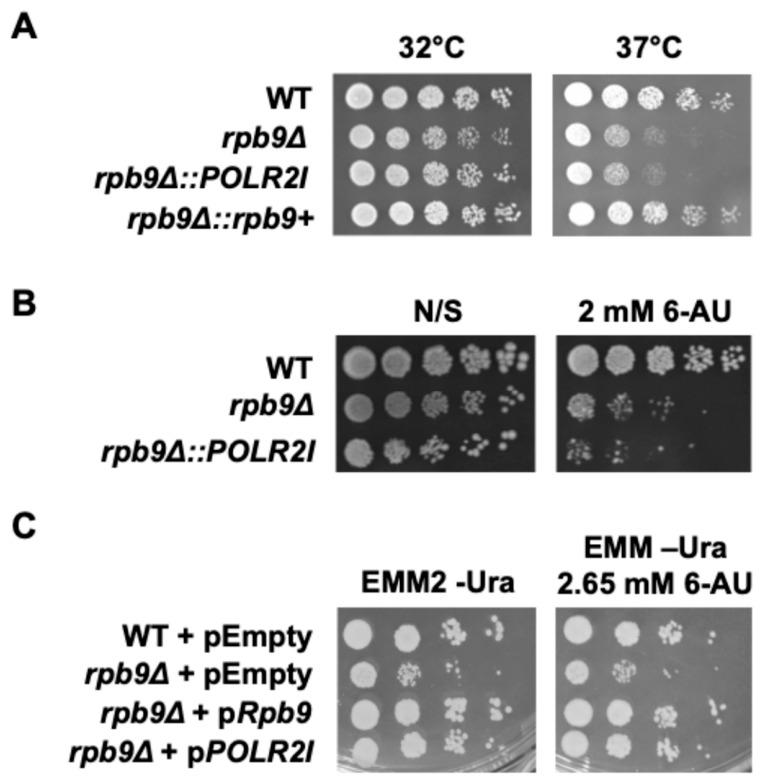
Endogenous *POLR2I* does not complement heat stress and 6-AU defects in our humanization system. (**A**) Spotting assays with serial dilutions of cells on non-selective (N/S) YEA media and grown at 32 °C or 37 °C. (**B**) Spotting assays at 32 °C with serial dilutions of cells on non-selective (N/S) YEA media or YEA media with 2 mM lithium chloride. For the assays shown in panels A and B, yeast strains with endogenous *rpb9*, *POLR2I*, or *rpb9Δ* were used. (**C**) Spotting assay at 32 °C using cells with empty (pEmpty) or expression plasmids (p*Rpb9*, p*POLR2I*). Media lacking uracil was used to maintain selection of the plasmids, therefore ensuring that the cells retain them.

One of the earliest reported phenotypes of *rpb9Δ* in the budding yeast *S. cerevisiae* was hypersensitivity to 37 °C elevated heat stress. We confirmed that this was also the case for *rpb9Δ* in *S. pombe* cells (Figure 5A) [17]. Interestingly, *POLR2I* in our humanization system could not complement the growth defect at 37 °C.

Yeast Rpb9 promotes transcription elongation at transcription pause/arrest sites [19,20]. Therefore, we tested whether *rpb9* and *POLR2I* are responsive to the transcription elongation inhibitor drug 6-azauracil (6-AU), which reduces transcription elongation by depleting intracellular levels of uracil triphosphates and guanine triphosphates [68]. After knocking out *rpb9*, we found that cell growth was strongly inhibited by 6-AU (Figure 5B). Our finding agrees with a previous study, which showed that *rpb9* loss in the budding yeast *S. cerevisiae* reduced cell growth on media containing 6-AU [19]. Sensitivity to 6-AU was *rpb9*-dependent because reintroduction of *S. pombe rpb9* (*rpb9Δ::rpb9+*) led to restored yeast growth on YEA media with 6-AU (Figure S4). In contrast, *rpb9Δ::POLR2I* cells grew poorly in the presence of 6-AU (Figure 5B). Therefore, endogenous *POLR2I* in our humanized system is unable to restore growth upon *rpb9* loss and 6-AU exposure. Whether the lack of complementation by *POLR2I* is related to transcription elongation remains unknown.

Next, we examined whether ectopic *POLR2I* could restore the growth of 6-AU-exposed *rpb9Δ* cells. Using our previously described plasmid-based approach to express *POLR2I*, from a plasmid with the P81*nmt1* promoter, in *rpb9Δ* cells [17,34], we found that ectopic *POLR2I* could complement 6-AU-associated growth defects (Figure 5C). In these cells, we previously showed that Rpb9 and POLR2I proteins are ectopically expressed at similar levels [17]. This suggests that the design of cross-species gene complementation assays can impact the levels of complementation.

### 3.6. Structural Similarities and Divergences of S. pombe Rbp9 and Human POLR2I

Protein sequence and structure are major determinants of protein functions [69]. Hence, the ability of endogenously expressed *POLR2I* to fully or partially complement many phenotypic roles of *S. pombe rpb9* suggests a high degree of conservation at the sequence and/or structural levels [17,70]. Indeed, the two homologs show 48.54% identity at the DNA sequence level and 48.21% identity at the amino acid level, based on the native sequences of *rpb9*/Rpb2 and *POLR2I* (Figure S5A,B). We leveraged AlphaFold to predict structures of *S. pombe* Rpb9 and human POLR2I (Figure 5A). Structural comparisons of the predicted models revealed high overall similarity. Since experimentally resolved structures of Rpb9 and POLR2I already exist [12,71], we additionally compared those empirical structures (Rpb9, 3H0G-I; POLR2I, 9EHZ-F) and also found high overall resemblance (Figure 5B). The TM-score, a measure of protein structure similarity [72], of Rpb9 and POLR2I is 0.84 (for the AlphaFold models) and 0.79 (for the empirical models). These scores suggest high overall similarity of Rpb9 and POLR2I. Lastly, we used AlphaFold to predict how POLR2I could interact with the two largest subunits of Pol II, Rpb1 and Rpb2, because structural features or interactions within the larger complex may influence the functions of Rpb9 and POLR2I [58]. We focused on Rpb1 and Rpb2 because Rpb9 primarily makes direct contact with those Pol II subunits [12]. The AlphaFold-generated models revealed notable overlaps in the predicted interactions of Rpb9 (or POLR2I) with *S. pombe* Rpb1 and Rpb2 (Figure 5C and Figure S5C,D). The α-carbon root mean square deviation (Cα RMSD) between the models is 0.61 angstroms (Å), indicating very high similarity between the two models [73]. Upon closer inspection, two structural differences were apparent. The first was at the N-terminus of POLR2I, which has an extended tail that is absent from Rpb9 (Figure S5C). Second, there was a small insertion in the second zinc finger domain of POLR2I that maps to residues 104–105 of Rpb9 (Figure S5C). Additionally, inspection of Rpb9 from the empirically resolved structure of *S. pombe* Pol II (PDB: 3H0G) revealed several amino acid residues that are within 5 Å of Rpb1 (Rpb9-N5, N52, E74, and R91) or Rpb2 (Rpb9-K42, R45, E47, M96, and Q113) and might mediate Rpb9-Rpb1 or Rpb9-Rpb2 interactions, but are not conserved in POLR2I (Figure S6). Future work will be required to determine whether these residues or structural differences could contribute to functional complementation differences between *S. pombe* Rpb9 and human POLR2I. Overall, our cross-species structural analyses suggest that Rpb9 and POLR2I are highly conserved at the sequence, structure, and function levels, albeit with noticeable structural differences that could be phenotypically significant. Our conclusions based on empirical and predicted structures are overall consistent with those of prior studies that used experimentally resolved structures of Rpb9 and POLR2I [17,32,34].

## 4. Discussion

Here, we developed a novel approach to humanize *S. pombe* yeast cells to investigate the functional conservation of yeast *rpb9* and its human homolog *POLR2I*. Our approach of gene swapping directly at the genomic *rpb9* locus represents an orthogonal cross-species gene complementation strategy compared to the conventional plasmid-based approach [17,34]. We find that *S. pombe rpb9* promotes general yeast growth, growth in the presence of various environmental stressors, and histone methylation at certain facultative heterochromatin loci. While our humanization system reveals that *POLR2I* can fully or partially complement most of the defects observed in *rpb9Δ* yeast cells, *POLR2I* was unable to restore cell growth in the presence of the 6-AU drug. However, this was context-dependent since plasmid-based *POLR2I* could rescue yeast sensitivity to 6-AU. Overall, our study corroborates prior conclusions from other studies that the functions of *rpb9* and *POLR2I* are mostly conserved. Moreover, it reveals that the functional conservation of these two genes can vary depending on the phenotype or the assay design, suggesting context-dependency for at least some of their functions.

We observed endogenous nuclear POLR2I protein levels to be markedly reduced when compared to Rpb9 levels. This was unexpected since *rpb9* and *POLR2I* were controlled by the same promoter, 5′- and 3′-UTRs, and presumably shared the same genomic context. Furthermore, the DNA sequence of the *POLR2I* ORF was codon-optimized for *S. pombe* expression. We propose that there are inherent differences in how Rpb9^POLR2I^ proteins are synthesized or stabilized in *S. pombe* cells. One possibility is that *POLR2I* is differently regulated at the transcriptional, post-transcriptional, or translational level(s). Another possibility is that the POLR2I protein is less stable compared to Rpb9, perhaps due to the absence of a human-specific stabilizing factor. These options may relate to the *POLR2I* introns that we intentionally omitted in our study design. Introns can enhance gene expression by affecting transcription, RNA transport, and stability, or translation [74,75]. *S. pombe rpb9* naturally has four introns [70], while *POLR2I* has five introns. Since *rpb9* in our wild-type and rescue strains contains its introns, but *POLR2I* lacks them, those introns could be important for enhancing the expression of *POLR2I*. Our unexpected finding should spur future studies into the potential role of *rpb9^POLR2I^* introns, or other factors, in regulating *POLR2I* expression. Given that the *POLR2I* copy number or expression is associated with human diseases [25,26], insights into how it is regulated would be informative from a human biology standpoint.

We found that *rpb9* and *POLR2I* share novel roles in H3K9 methylation at the facultative heterochromatin loci *mei4* and *ssm4*. This suggests that both proteins may have additional roles in chromatin modifications, perhaps in addition to (or as a consequence of) transcription and transcription-coupled DNA repair [19,21]. Interestingly, H3K9me2 at these loci requires the *S. pombe* RNA-binding protein Mmi1 [57], which we previously found to directly interact with *S. pombe* Rpb9 [58]. Also, transcription of the *ssm4* locus is required for H3K9me2 formation at that region [57]. It is possible that *rpb9* and *POLR2I* promote Mmi1-dependent H3K9me2 indirectly through conserved transcription initiation and/or elongation functions [18,20]. Transcription would generate nascent RNAs that Mmi1 [76] could bind with to begin the H3K9 methylation process [57,59,60,77]. Alternatively, Rpb9^POLR2I^ could directly recruit or stabilize Mmi1 at the *mei4* and *ssm4* loci to promote H3K9 methylation. As *S. pombe* Mmi1 and its mouse homolog YTHDC1 [78] repress genes, in part, through H3K9 methylation [57,79], future studies into how Rpb9^POLR2I^ could be involved would improve our understanding of how RNA-binding proteins coordinate with Pol II and RNAs to regulate chromatin modifications.

**Figure 6:**
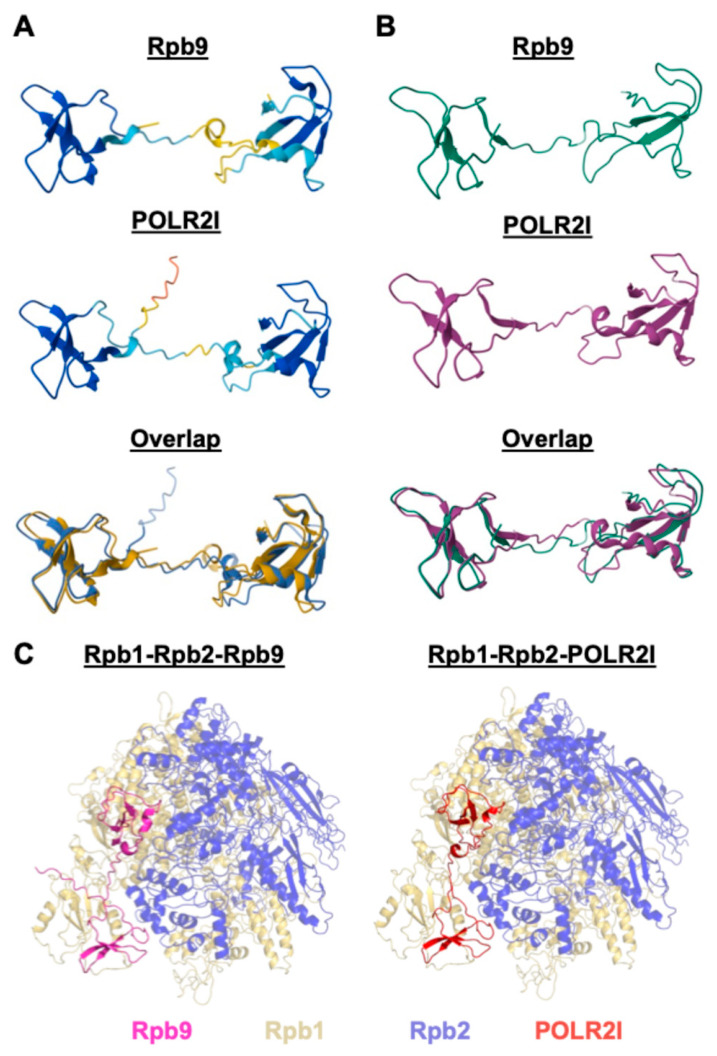
Structural analyses of Rpb9 and POLR2I. (**A**) AlphaFold-predicted structures of *S. pombe* Rpb9 (top) and human POLR2I (middle). Both structures are color-coded based on pIDDT confidence scores with blue (>90): backbone and sidechain are accurate; teal (90 to 70): backbone good, side chain inaccurate; yellow (70 to 50): possible backbone inaccuracy; and orange (<50): flexible spot or intrinsically disordered. At the bottom, the structures are overlaid, with Rpb9 in dark yellow and POLR2I in blue. (**B**) Empirically resolved structures of *S. pombe* Rpb9 (top, PDB: 3H0G-I), human POLR2I (middle, PDB: 9EHZ-F), and overlaid (bottom). For the overlaid bottom structure, green is Rpb9 and magenta is POLR2I. (**C**) AlphaFold-predicted structure of *S. pombe* Rpb9 interacting with *S. pombe* Rpb1 and Rpb2 (**left**) and AlphaFold-predicted structure of human POLR2I interacting with *S. pombe* Rpb1 and Rpb2 (**right**).

Of all the phenotypes that we tested, endogenously expressed *POLR2I* failed to complement *rpb9*-dependent 37 °C and 6-AU hypersensitivity in our yeast system. However, reintroducing endogenous *S. pombe rpb9+* or ectopically expressed *POLR2I* restored growth. One possible explanation for these non-complementations is that there is not enough expression of functional POLR2I in our endogenous system (Figure 1B,C). In support of this model, it was previously shown in *S. cerevisiae* that POLR2I expression from a high-copy plasmid could restore growth at 37 °C, but not if expressed from a low-copy plasmid [32]. Additionally, we observed complementation of *S. pombe* yeast growth in the presence of 6-AU when POLR2I was ectopically expressed (Figure 5C). It was previously shown that the POLR2I protein level, in this ectopic system, was similar to that of ectopically expressed Rpb9 in *S. pombe* cells [17]. It remains unknown whether the levels of POLR2I in the ectopic system match (or exceed) the level of Rpb9 protein in the endogenous system. Altering the ectopic system to permit POLR2I expression in non-selective YEA media will be required to experimentally compare protein levels between the two expression systems. A second possible explanation for the non-complementation cases is that *rpb9* and *POLR2I* might have divergent roles in the complementable and non-complementable phenotypes. These roles may include yeast- and human-specific mechanisms that are affected by the sequence and/or structural differences between Rpb9 and POLR2I (Figure 6C, Figures S5C and S6). For example, the extra N-terminal tail of POLR2I, which is absent in Rpb9, might influence 6-AU responses differently. Our lack of complementation finding is important because it reveals a phenotype that is distinct from the others, which is genetic support for the notion that *rpb9^POLR2I^* may be multifunctional with distinct roles [20]. Whether the role of *rpb9* in 6-AU response is generally related to transcription elongation or specifically to the 6-AU compound requires further investigation. Future studies could examine the interaction of *rpb9^POLR2I^* with genetic mutants that perturb transcription elongation.

The only other studies of functional complementation between *S. pombe rpb9* and human *POLR2I* were performed by us [17,34]. Although both our previous and current work provide largely consistent evidence for functional conservation between these homologs, we observed a discrepancy in growth responses to 6-AU. These findings underscore the value of employing orthogonal experimental approaches to dissect distinct determinants of functional conservation. While successful complementation demonstrates that homologous proteins can fulfill similar molecular roles, divergent complementation outcomes across experimental designs (such as those observed for 6-AU sensitivity) suggest that cellular and regulatory context influences how these conserved functions are executed.

## Figures and Tables

**Table 1:** *S. pombe* growth phenotypes during our procedure to endogenously replace *rpb9* for *POLR2I*.

Genotype	Expected Growth in the Presence of G418 Drug ^1^	Expected Growth in the Presence of 5-FOA Drug ^2^
*rpb9+*	Not viable	Viable
*rpb9Δ::ura4-kanMX*	Viable	Not viable
*rpb9Δ::POLR2I*	Not viable	Viable

^1^ *S. pombe* cells expressing the *kanMX* gene are resistant to the G418 antibiotic [40]. ^2^ Yeast cells expressing the *ura4* gene are sensitive to compound 5-fluoroorotic acid (5-FOA) [38,41].

## Data Availability

The yeast strains and plasmids that were used for this work are available from the corresponding author.

## Supplementary Materials

The following supporting information can be downloaded at: https://www.mdpi.com/article/10.3390/genes17060606/s1, Table S1. Yeast strains used in this study. Table S2. DNA sequence of POLR2I used in this study. Table S3. Oligos used in this study. Table S4. OD_600_ measurements of yeast strains (N = 3) that were grown in YEA media over 27 h at 32 °C. Figure S1. PCR-based validation of yeast strain genotypes. Figure S2. Yeast colony sizes on non-selective YEA media. Figure S3. Reintroduction of *rpb9* rescues *rpb9Δ* defects. Figure S4. Reintroduction of *rpb9* rescues yeast growth on media with 6-AU. Figure S5. DNA and amino acid sequence alignments of *S. pombe rpb9*/Rpb9 and native consensus human *POLR2I*/POLR2I. Figure S6. Rpb9 residues that could interact with *S. pombe* Rpb1 or Rpb2, but are not conserved in POLR2I.

## Abbreviations

The following abbreviations are used in this manuscript:

Pol II	RNA polymerase II
ORF	Open reading frame
UTR	Untranslated region
cDNA	Complementary DNA
YEA	Yeast extract with adenine and glucose
PMG	Pombe minimal glutamate
qPCR	Quantitative polymerase chain reaction
ChIP	Chromatin immunoprecipitation
H3K9me2	Di-methylation of histone H3 at lysine 9
5-FOA	5-fluoroorotic acid
5-FU	5-fluorouracil
6-AU	6-azauracil
MPA	Mycophenolic acid
TBZ	Thiabendazole
*S. cerevisiae*	*Saccharomyces cerevisiae*
*S. pombe*	*Schizosaccharomyces pombe*
*rpb9Δ*	Endogenous deletion of the *rpb9* gene
*rpb9Δ::rpb9+*	Endogenous *rpb9* swapped for *rpb9*
*rpb9Δ::POLR2I*	Endogenous *rpb9* swapped for *POLR2I*
