# Supplementary material for: Humanization of the rpb9 Locus in Fission Yeast Reveals Conserved and Divergent Roles of rpb9 and Human POLR2I
Source: Genes (Basel). 2026 May 27;17(6):606. doi: 10.3390/genes17060606 (PMC13300194; doi:10.3390/genes17060606)
Supplement: Supplementary file 1 [file genes-17-00606-s001.zip › Supplementary_Figures.pdf]

Supplementary Figures

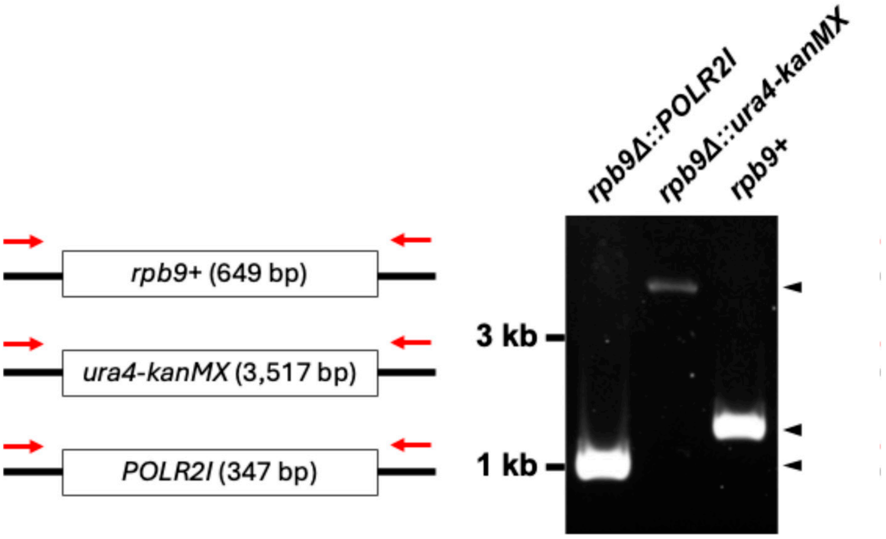

Figure S1. PCR-based validation of yeast strain genotypes. PCR validation of *rpb9*, *ura4-kanMX*, and *POLR2I* genes at the native *rpb9* locus in *S. pombe*. For each strain, the same set of PCR oligos was used, which targeted upstream and downstream of the swap region.

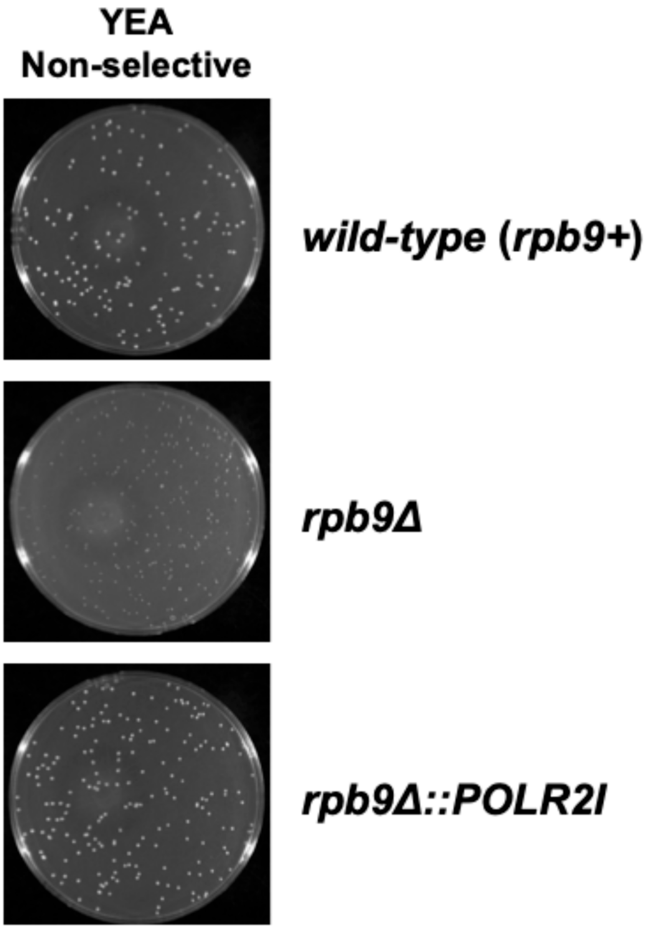

Figure S2. Yeast colony sizes on non-selective YEA media. Yeast colonies on non-selective YEA media plates. Approximately 1,000 yeast cells were beadspread onto the plates and incubated at 32°C for 3-5 days prior to imaging using a BioRad ChemiDoc imager.

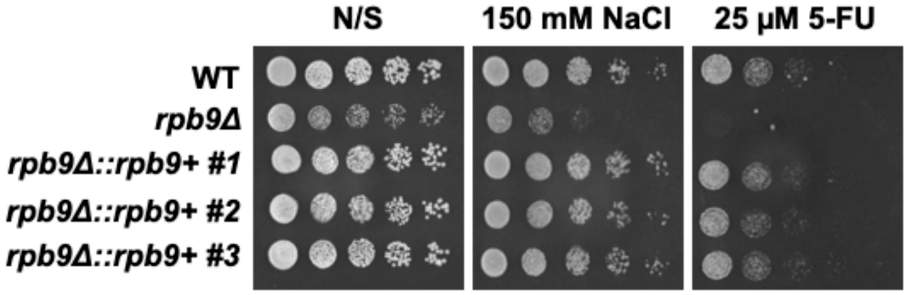

Figure S3. Reintroduction of *rpb9* rescues *rpb9Δ* defects. Spotting assays with YEA media -/+ 150mM NaCl or 25 μM 5-FU. Three independent isolate strains with re-integrated *rpb9+* back into the native *rpb9* locus was tested.

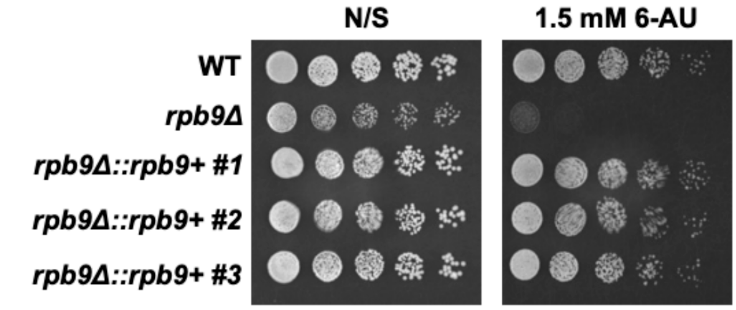

Figure S4. Reintroduction of *rpb9* rescues yeast growth on media with 6-AU. Spotting assays with YEA media -/+ 150mM NaCl or 1.5 mM 6-AU. The non-selective plate is identical to the one in Figure S3.

A

```
  1 -----------------------------------ATGTCAAATT---TTCAATATTGTATAGAATGCAATAATATGCTGTACCCTAGAGAGGATAAAGT     62
                                     ..||   |||   |||     ||....|||||.||.||.|||||||||||.|..||.||.||.|.
  1 ATGGAGCCCGACGGGACTTACGAGCCGGGCTTCGTGGGT---ATTCGCTTC-----TGCCAGGAATGTAACAACATGCTGTACCCCAAGGAAGACAAGGA     92

 63 GGATCGTGTTCTGAGGCTA-GCTTGTCGTAATTGTGATTACTCAGAGATCGCTGCTACTAGTAAAGTTTATCGCCATGAG--TTACAA-AGCTCCA---A    155
    |.|.||..||||| ..||| ||.||.||.||.||||||||| |||                             ||.|||  ..|||| ||||.||   |
 93 GAACCGCATTCTG-CTCTACGCGTGCCGGAACTGTGATTAC-CAG-----------------------------CAGGAGGCCGACAACAGCTGCATCTA    161

156 TGTAGAAAA---CACG-ACG--GT-------------CAG----TC-ACGACGCTTCAACGGACCCTACTTT-------ACCTAGATCAGACAAAGAATG    224
    |||..|.||   |||| |||  ||             |||    || .|||||..||...||||||.||.||       |||.||    |||.|
162 TGTCAACAAGATCACGCACGAAGTGGACGAACTGACCCAGATTATCGCCGACGTGTCCCAGGACCCCACGTTGCCGCGGACCGAG----GACCA------    251

225 CCCGCGTTGTCA---------TCAACATGAAGCAGTTTTTTATCAG--ACTCATTCTCGT---CGGG----GTGATACTATGATGACTCTGATT--TATG    304
    ||||   ||.||         .||..|.||.||.||.||.|..|||  ||.||     ||   ||||    | ||..|.||| .|.||    ||  ||.|
252 CCCG---TGCCAAAAGTGCGGCCACAAGGAGGCTGTGTTCTTCCAGTCACACA-----GTGCGCGGGCCGAG-GACGCCATG-CGCCT----TTACTACG    337

305 TTTG------CGTACATTGTGG--ATTTGCTTTTGAAGAAC--AGTAA    342
    |.||      |..|||.||.||  | ..|||      |.||  |||.|
338 TGTGCACAGCCCCACACTGCGGCCA-CCGCT------GGACCGAGTGA    378
```

B

```
  1 ----------MSNFQYCIECNNMLYPREDKVDRVLRLACRNCDYSEIAATSKVYRHELQSSNVENTTVSHDASTDPTLPRS-DKECPRCHQHEAVFYQTH     89
              ....::|.||||||||:|||.:|:|..|||||||.:.|..|.:|.:::.....|.|.:..|.|.||||||: |..|.:|...||||:|:|
  1 MEPDGTYEPGFVGIRFCQECNNMLYPKEDKENRILLYACRNCDYQQEADNSCIYVNKITHEVDELTQIIADVSQDPTLPRTEDHPCQKCGHKEAVFFQSH    100

 90 SRRGDTMMTLIYVCV--HCGFAFEEQ    113
    |.|.:..|.|.|||.  |||..:.|
101 SARAEDAMRLYYVCTAPHCGHRWTE-    125
```

C

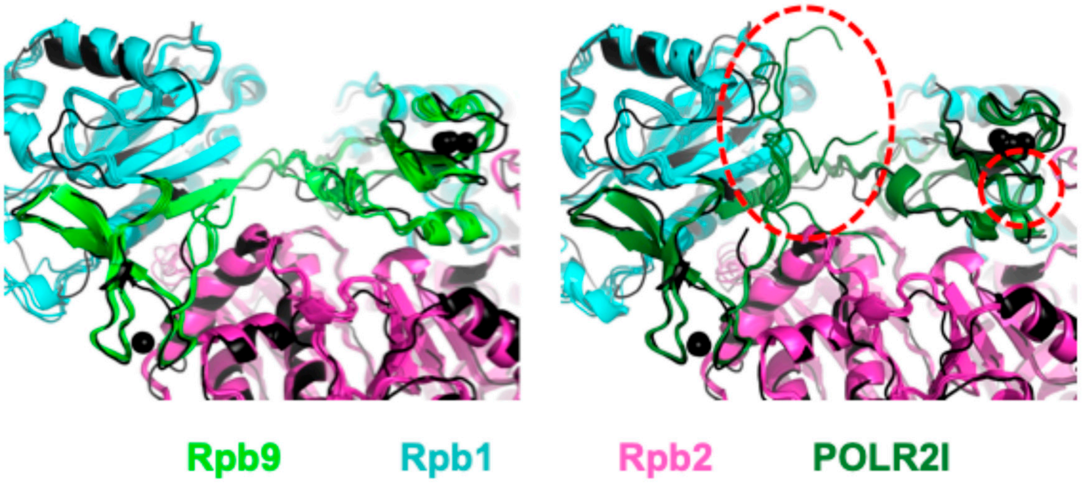

D

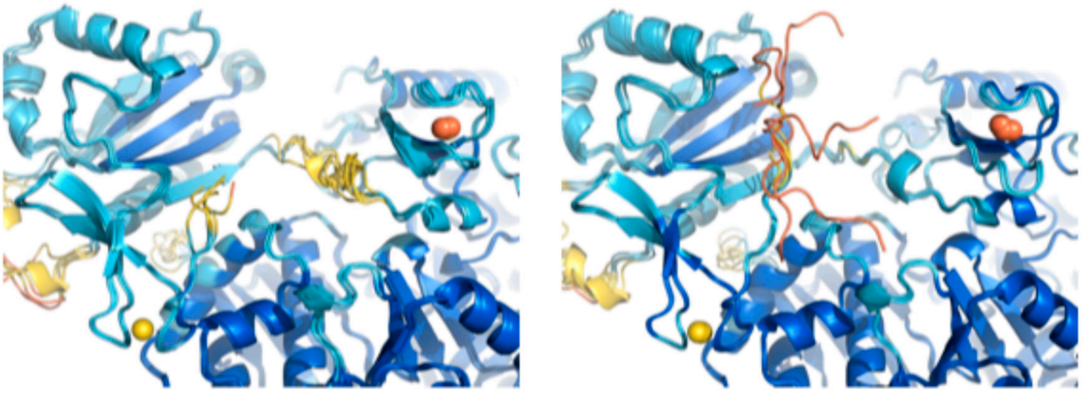

Figure S5. DNA and amino acid sequence alignments of *S. pombe* Rpb9 and native consensus human POLR2I. (**A**) Alignment of the DNA sequences of the open reading frames of *S. pombe rpb9* and human *POLR2I*. (**B**) Alignment of the amino acid sequences of *S. pombe* Rpb9 and human POLR2I. (**C**) (Left) The five AlphaFold3 models for the Rpb1/2/9 complex are shown with Rpb1 in cyan, Rpb2 in magenta and Rpb9 in green. The models were overlaid with the experimentally determined structure of *S. pombe* Rpb1-Rpb2-Rpb9 (black, PDB 3H0G). Black spheres indicate Zn^{2+} ions. The $C\alpha$ RMSD for the top AlphaFold3 model and 3H0G structure is 1.31, indicating very close structural similarity. (Right) Five AlphaFold3 models of Rpb1/2/POLR2I, with Rpb1 in cyan, Rpb2 in magenta, and POLR2I in dark green. Dashed red circles highlight structural differences between Rpb9 and POLR2I within those AlphaFold3 models. The N-terminus of POLR2I is likely disordered. (**D**) (Left) AlphaFold3 model of the Rpb1/2/9 complex in Fig. S5C (left) as colored based on pLDDT scores using the AlphaFold convention. (Right) AlphaFold3 model of the Rpb1/2/POLR2I complex in Fig. S5C (right) as colored based on pLDDT scores using the AlphaFold convention. The color convention is navy blue (pLDDT > 90, very high confidence), cyan (90 > pLDDT > 70, high confidence), yellow (70 > pLDDT > 50, low confidence), and orange (pLDDT < 50, very low confidence). Zn^{2+} ions are shown as spheres and are colored based on pLDDT scores.

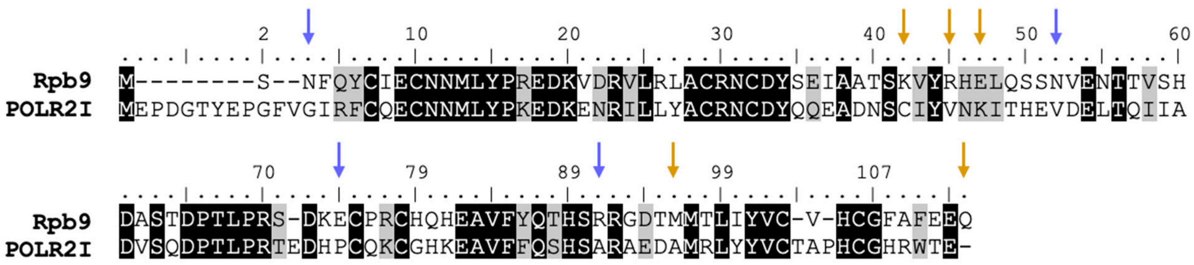

Figure S6. Non-conserved Rpb9 residues that could interact with *S. pombe* Rpb1 or Rpb2. From the resolved structure of *S. pombe* Pol II (PDB: 3H0G), resides of *S. pombe* Rpb9 were identified that were within 5 angstroms away from Rpb1 or Rpb2. Gold arrows point to identified residues that were near Rpb1 and periwinkle arrows point to identified residues that were near Rpb2. All arrows point to residues that are not conserved between Rpb9 and POLR2I.
